# Genome editing with the donor plasmid equipped with synthetic crRNA-target sequence

**DOI:** 10.1038/s41598-020-70804-6

**Published:** 2020-08-24

**Authors:** Riki Ishibashi, Kota Abe, Nanami Ido, Satsuki Kitano, Hitoshi Miyachi, Fumiko Toyoshima

**Affiliations:** 1 Department of Biosystems Science, Institute for Frontier and Medical Sciences, Kyoto University, Sakyo-ku, Kyoto, 606-8507 Japan; 2 Department of Mammalian Regulatory Networks, Graduate School of Biostudies, Kyoto University, Sakyo-ku, Kyoto, 606-8502 Japan

**Keywords:** Genetic engineering, CRISPR-Cas9 genome editing

## Abstract

CRISPR/Cas-mediated genome editing is a powerful tool for generating genetically mutated cells and organisms. Linearisation of donor cassettes with this system has been shown to facilitate both transgene donor insertion and targeted knock-in. Here, we developed a donor plasmid that we name pCriMGET (plasmid of synthetic CRISPR coded RNA target sequence-equipped donor plasmid-mediated gene targeting), in which an off-target free synthetic CRISPR coded RNA-target sequence (syn-crRNA-TS) is incorporated with a multi-cloning site, where a donor cassette can be inserted. With co-expression of Cas9 and the syn-crRNA-TS guide RNA (gRNA), pCriMGET provides a linearised donor cassette in vivo, thereby promoting the transgene donor insertion and targeted knock-in. When co-injected with Cas9 protein and gRNA into murine zygotes, pCriMGET yielded around 20% transgene insertion in embryos. This method also achieved more than 25% in-frame knock-in at the mouse *Tbx3* gene locus without predicted insertion–deletion mutations using a transgene donor with 400-bp homology arms. pCriMGET is therefore useful as a versatile CRISPR/Cas9-cleavable donor plasmid for efficient integration and targeted knock-in of exogenous DNA in mice.

## Introduction

The generation of genetically mutated animals is essential for studies of gene function and pathological analysis in vivo^1,2^. The genome editing methods using site-specific nucleases, including zinc-finger nucleases (ZFNs), transcription activator-like nucleases (TALENs) and RNA-guided nucleases, have made it possible to integrate exogenous DNA into targeted genomic loci^3^. In particular, the clustered regularly interspaced short palindromic repeats (CRISPR)/CRISPR-associated (Cas) system has been widely used because of its simple procedures and high versatility in targeting genomic loci. Using a guide RNA (gRNA) for a target sequence, CRISPR/Cas induces double-strand breaks (DSBs) in the target genomic locus, at which exogenous DNA can be integrated via DNA repair pathways. Because the gene targeting efficiency of the conventional method based on the homologous recombination (HR) pathway is low^4,5^, substantial efforts have been made to develop efficient and precise targeted knock-in methods. The strategies based on the repair pathways of nonhomologous end-joining (NHEJ) and microhomology-mediated end-joining (MMEJ) have achieved targeted transgene integration in zebrafish and mice with no or short homology arms on both sides of the donor cassette^6–13^. However, these methods often cause undesired insertion–deletion (indel) mutations at the DSB sites^6–13^. Other groups reported the Easi-CRISPR targeting method for the generation of knock-in mice using long single-stranded DNA (ssDNA) as a donor template^14,15^. However, this method has a limitation in terms of the donor size (< 2 kb) and is prone to cause rearranged alleles including indels^16^. The Tild-CRISPR method, in which PCR-amplified or in vitro cleaved linearised double-stranded DNA is using as a donor, exhibits high knock-in efficiency in mice^17^. In addition, recent reports have proposed in vivo cleavable donor plasmids, in which single guide (sg)RNA-targeting sequences sandwich the donor cassette, which enables Cas9/sgRNA complex to induce DSBs at the target genomic locus and at both ends of the donor cassette on the donor plasmid simultaneously^18,19^. This homology-mediated end-joining (HMEJ)-based method has also yielded highly efficient gene targeting, but there is a need to add sgRNA-targeting sequences at both ends of the donor cassette in every donor plasmid.

Here, we report a new type of in vivo cleavable donor plasmid, in which synthetic CRISPR coded RNA-target sequences (syn-crRNA-TS) with no off-target potential sandwich the multi-cloning site (MCS). By in vivo linearisation of the transgene donor with Cas9/syn-crRNA-TS gRNA, this method achieved highly efficient transgene insertion and in-frame knock-in in mice.

## Results

### Generation of pCriMGET

**Figure 1:**
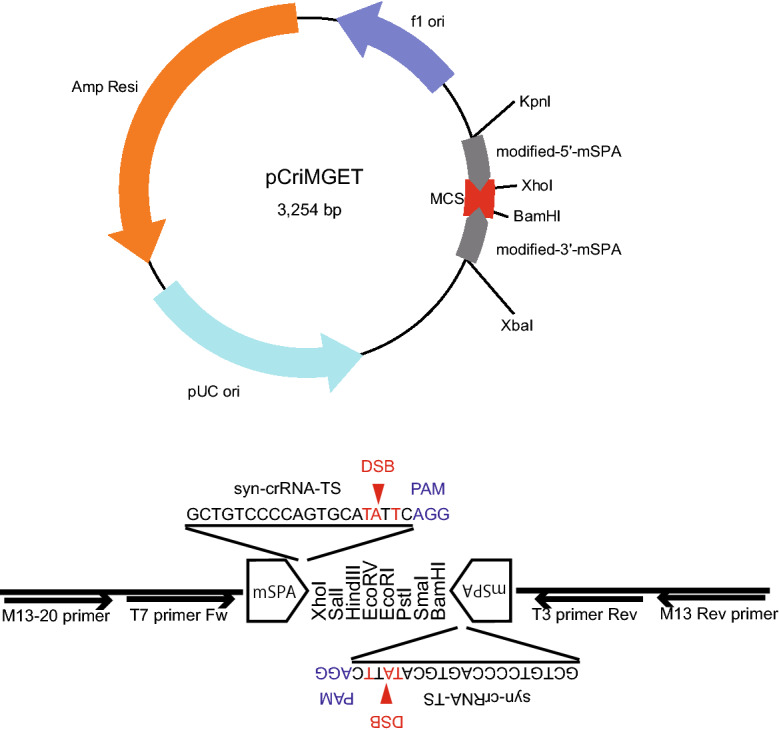
Schematic of pCriMGET. pCriMGET, based on pBluescriptII SK(+), harbours mSPAs at the 5′- and 3′-ends of MCS. The modified nucleotides in SPA and PAM sequences are shown in red and blue, respectively. The syn-crRNA-TS is underlined. The predicted DSB sites within syn-crRNA-TS are shown by red arrowheads. Black arrows show M13-20, T7, T3 and M13 universal primers. Restriction enzyme recognition sites within MCS are shown.

To design syn-crRNA-TS with minimal off-targeting potential in mice and humans, we chose a synthetic poly(A) site (SPA) of the rabbit β-globin gene^20,21^, which harbours seven putative crRNA-TSs that have no match to any region in the mouse genome (Fig. S1A,B). Among them, four crRNA-TSs, which have no T-rich sequences and partially overlap within positions 115–144 on the forward (+) and reverse (−) strands, share the same sequence with only one region in the human genome (Fig. S1B). To reduce off-targeting potential in humans, three nucleotide mutations were introduced in the crRNA-TSs at positions 115–137 in SPA, so that no matched sequences were present in both human and mouse genomes (Fig. S1A,B). Then, the MCS flanked by the syn-crRNA-TSs was inserted into pBluescriptII SK(+). We named the obtained vector pCriMGET (plasmid of synthetic CRISPR coded RNA target sequence-equipped donor plasmid-mediated gene targeting) (Fig. 1). By incorporating the donor cassette into MCS, pCriMGET is expected to be ready for use as a CRISPR/Cas9-cleavable donor plasmid with no need to design crRNA target sites at both ends of the donor cassette.

### Intracellular cleavage of pCriMGET by CRISPR/Cas9

**Figure 2:**
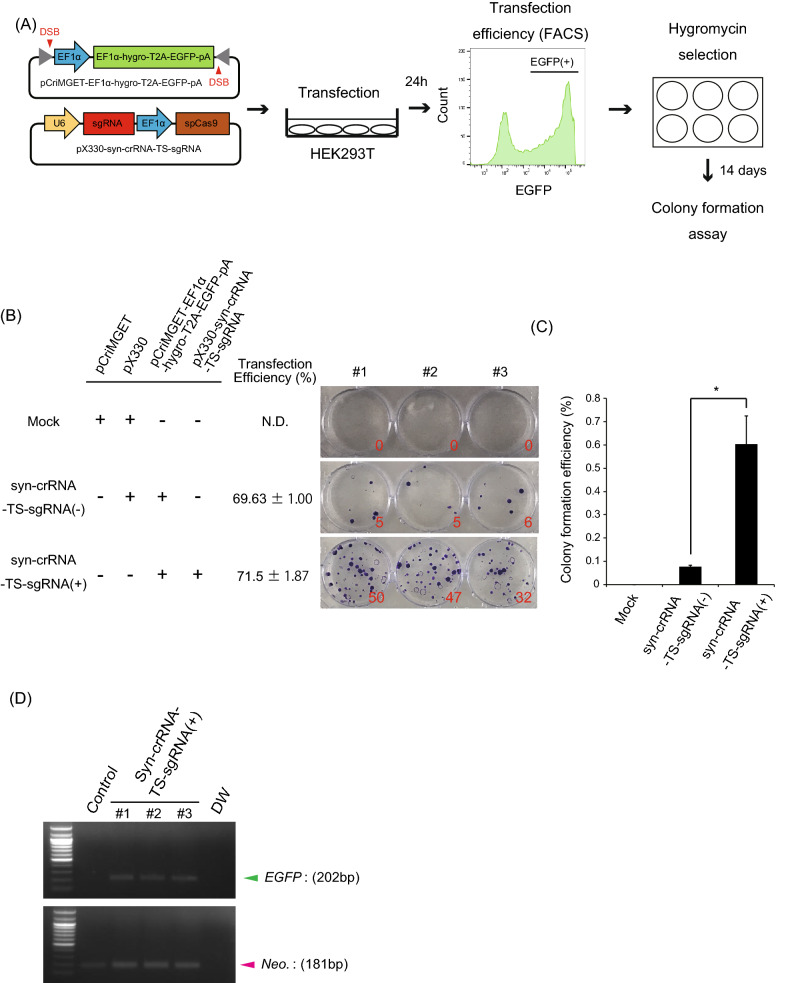
Intracellular cleavage of pCriMGET by CRISPR/Cas9. (**A**) A flowchart of the experiments for analysing the efficiency of transgene insertion via the pCriMGET system. (**B**) Hygromycin-resistant colony formation assay. Calculation of transfection efficiency and colony formation assay were performed at 24 h and 14 days after transfection, respectively. Colony numbers are shown in red letters. (**C**) Colony formation efficiency of each sample, indicating enhanced transgene insertion by the pCriMGET system. Mean ± s.d. from three experiments; *P < 0.05 analysed by the two-tailed t-test. (**D**) Analysis of EF1α-hygro-T2A-EGFP-pA cassette integration by PCR amplification of the *EGFP* gene. PCR amplification of the neomycin resistance gene was used as an internal control. spCas9: *S. pyogenes* Cas9. N.D.: not detected. DW: distilled water.

To test whether CRISPR/Cas9 induces DSBs on pCriMGET at the syn-crRNA-TS in vivo, we transfected HEK293T cells with pCriMGET incorporated with the donor cassette encoding the *EF1α promoter-hygromycin resistant gene-T2A-EGFP-bovine growth hormone polyadenylation signal sequence (bGHpA)* (pCriMGET-EF1α-hygro-T2A-EGFP-pA), together with pX330^22^, a vector that expresses both humanised *Staphylococcus pyogenes* (sp) Cas9 protein and syn-crRNA-TS targeting sgRNA (pX330-syn-crRNA-TS-sgRNA) (Fig. 2A). After analysing the transfection efficiency by EGFP expression using flow cytometry at 24 h, cells were selected using hygromycin for 14 days. Because linearisation of the donor plasmid promotes integration of the transgene into the host genome, pX330-syn-crRNA-TS-sgRNA is expected to increase the genomic integration efficiency of the transgene donor, thereby promoting hygromycin-resistant colony formation. We found that the efficiency of colony formation was significantly increased when pCriMGET-EF1α-hygro-T2A-EGFP-pA was co-transfected with pX330-syn-crRNA-TS-sgRNA, compared with pX330 vector co-transfection (Fig. 2B,C). In addition, we confirmed genomic integration of the donor cassette (EF1α-hygro-T2A-EGFP-pA) in cells (Fig. 2D). These results indicate that CRISPR/Cas9 induces DSBs on pCriMGET at the syn-crRNA-TS in vivo.

### Precise in-frame knock-in of exogenous DNA by pCriMGET in culture cells

**Figure 3:**
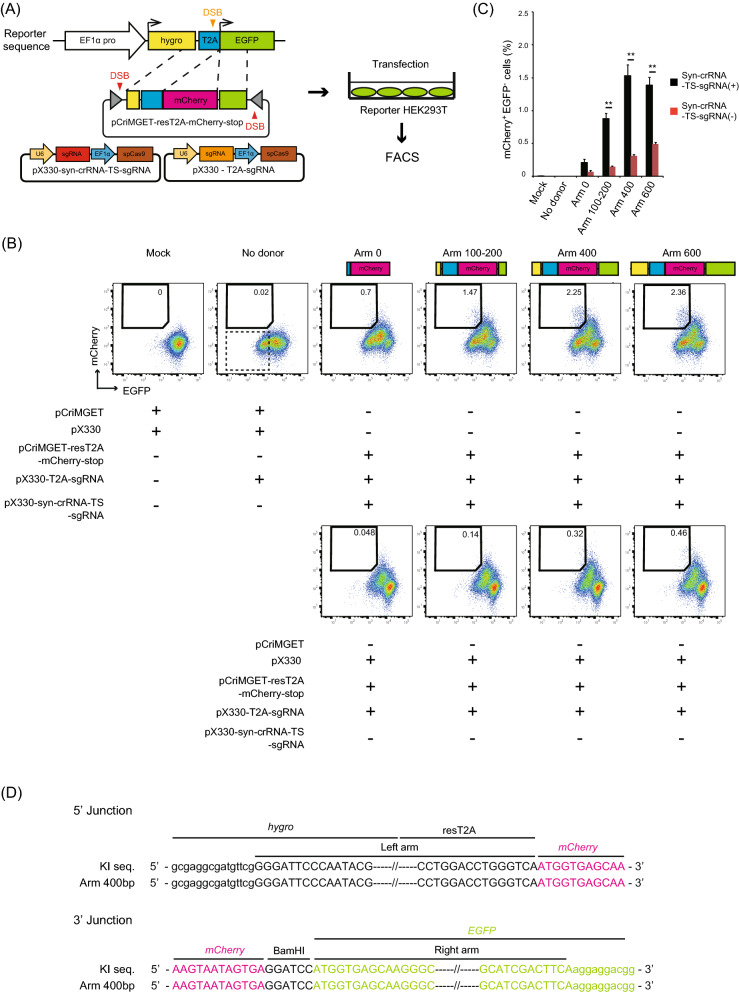
Precise in-frame knock-in of exogenous DNA via pCriMGET system in mCherry/EGFP-HDR reporter system. (**A**) Strategy of the mCherry/EGFP-HDR reporter system for analysing in-frame knock-in efficiency of the pCriMGET system. (**B**) FACS analysis of mCherry/EGFP expression in the mCherry/EGFP-HDR reporter cells transfected with the indicated plasmids. The percentages of mCherry^+^EGFP^−^ cells are shown in each gate. The EGFP^−^ cell population that emerged in the no donor sample is delineated with a dashed line. (**C**) Average percentages of mCherry^+^EGFP^−^ cells in each sample. Mean ± s.d. from three experiments. **P < 0.01 analysed by Tukey’s multiple comparison tests. (**D**) Sequence analysis of 5′- and 3′-junction regions of targeting locus. Upper- and lower-case letters indicate sequences inside and outside of the donor cassette, respectively. The results of a sample using pCriMGET-resT2A-mCherry-stop-Arm400 are shown.

We next examined whether pCriMGET can be applied for in-frame knock-in of exogenous DNA within culture cells. To this end, we established an mCherry/EGFP-homology-directed repair (HDR) reporter system, which monitors the in-frame knock-in of transgene donors into target genomic loci by mCherry/EGFP conversion (Fig. 3A), in accordance with the mCherry-HDR reporter system described previously^18^. First, we established a reporter HEK293T single-cell clone in which *hygro-T2A-EGFP* was genomically integrated (Fig. S2). We then constructed pCriMGET-resT2A-mCherry-stop, which incorporates the donor cassette encoding *sgRNA resistant T2A-mCherry-3x stop codons*, flanked by homology arms (Fig. 3A). To optimise the length of the homology arms, the donor cassette was flanked by homology arms of various lengths (Fig. 3B). The reporter HEK293T cells were transfected with pCriMGET-resT2A-mCherry-stop, together with pX330-syn-crRNA-TS-sgRNA and pX330-T2A-sgRNA, which are expected to induce DSBs on pCriMGET at syn-crRNA-TS and on the genomically integrated T2A site, respectively (Fig. 3A). The cleavage at the T2A site was confirmed by the emergence of an EGFP^−^ cell population transfected with pX330-T2A-sgRNA alone, compared with mock-transfected cells (Fig. 3B; no donor, indicated by a dashed line). When the cells were transfected with all three vectors, the mCherry^+^EGFP^−^ cell population was increased in a manner dependent on the length of the homology arms, with a plateau at 400 bp, but to a lesser extent in the absence of pX330-syn-crRNA-TS-sgRNA (Fig. 3B,C). The mCherry^+^EGFP^+^ cell population was increased in the transfected cells, which is presumably attributable to the multiple copy number of *hygro-T2A-EGFP* in the reporter HEK293T clone used in the assay and random integrations of the donor gene. Next, we sequenced the T2A site genomic region in transfected cells and confirmed knock-in of the donor gene (*mCherry*) with no indels or frame-shift in the 5′ and 3′ junction regions (Fig. 3D). These findings indicate that pCriMGET induced in-frame knock-in of the donor cassette with 400-bp homology arms.

**Figure 4:**
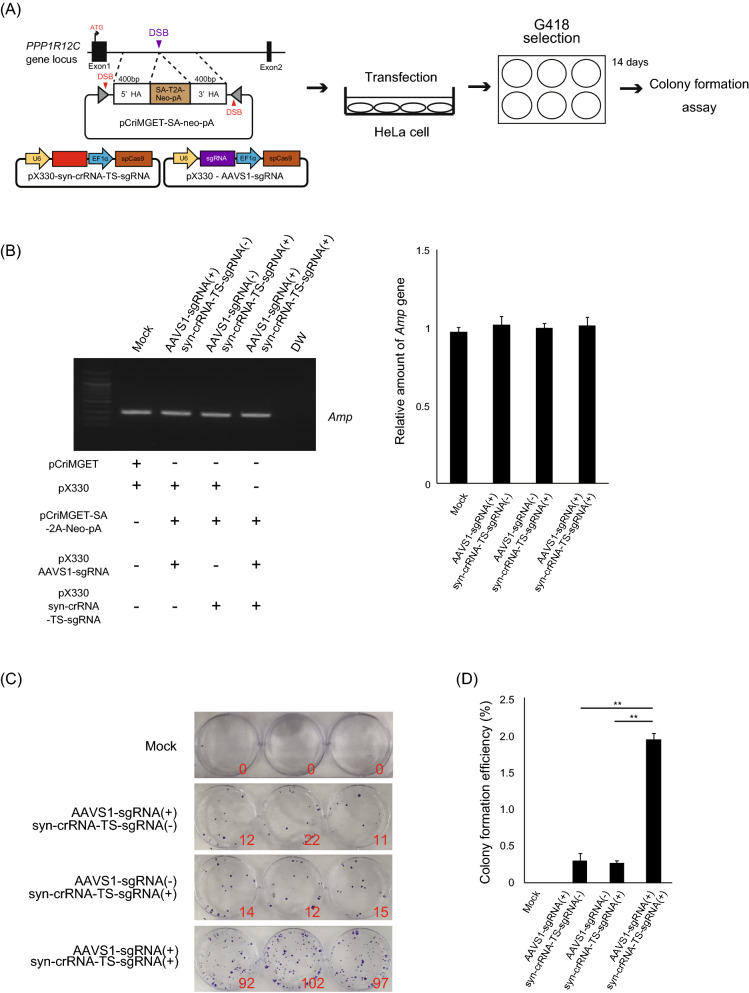
Precise in-frame knock-in of exogenous DNA via pCriMGET system in endogenous genomic region of culture cells. (**A**) Strategy of SA-T2A-Neo-pA knock-in into *AAVS1* within the *PPP1R12C* gene locus for analysing the in-frame knock-in efficiency of the pCriMGET system. (**B**) Analysis of transfection efficiency by PCR amplification of the *Amp* gene coded on pCriMGET and pX330 plasmids. The intensity of each band was measured by ImageJ software and normalised with mock sample. Relative amount of the *Amp* gene is shown on the right. Mean ± s.d. from three experiments. (**C**) G418-resistant colony formation assay. Colony numbers are shown in red letters. (**D**) Colony formation efficiency of each sample, indicating enhanced transgene knock-in into the *AAVS1* genomic locus via the pCriMGET system. Mean ± s.d. from three experiments; **P < 0.01 analysed by Dunnett’s multiple-comparison test.

We further examined whether the pCriMGET/pX330 system could be applied for transgene knock-in into the endogenous AAVS1 site, a well-known safe harbour within the human *PPP1R12C* gene locus. To this end, we constructed pCriMGET-SA-neo-pA, which incorporates the donor cassette encoding *AAVS1-sgRNA-targeting site-harbouring splicing acceptor (SA)-T2A-neomycin resistance gene (neo)-bGHpA*, flanked by 400-bp homology arms (Fig. 4A). Then, we transfected HeLa cells with pCriMGET-SA-neo-pA, together with pX330-syn-crRNA-TS-sgRNA and pX330-AAVS1-sgRNA^23^, followed by G418 selection for 14 days. The colony formation efficiency was significantly increased when cells were transfected with all three vectors compared with that in the absence of pX330-AAVS1-sgRNA, pX330-syn-crRNA-TS-sgRNA or both (Fig. 4B–D). These results indicate that the pCriMGET/pX330 system can induce in-frame exogenous transgene knock-in within culture cells via CRISPR/Cas9-mediated cleavage of syn-crRNA-TS.

### Efficient generation of transgenic mice with pCriMGET

**Figure 5:**
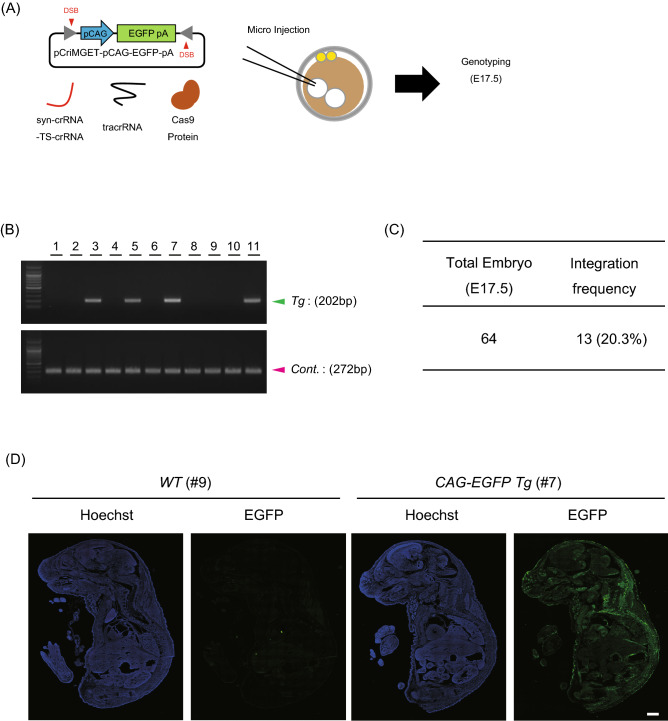
Generation of *CAG-EGFP* transgenic mice via pCriMGET system. (**A**) Strategy for the generation of *CAG-EGFP* transgenic mice via the pCriMGET system. (**B**) Genotyping PCR for *CAG-EGFP* transgenic mouse embryos at E17.5. Upper: PCR primer sets on the *pCAG-EGFP-pA* cassette yield a 202-bp band. Lower: PCR primer sets on the internal control genomic region on chromosome 8 yield a 272-bp band. Representative images are shown. (**C**) Integration frequency of the embryos. (**D**) Representative immunofluorescence images of *WT* and *CAG-EGFP* transgenic mouse embryos at E17.5. Hoechst (blue) and EGFP (green). Scale bar, 1 mm.

We further attempted to generate transgenic mice with pCriMGET. We constructed pCriMGET that incorporates the donor cassette encoding *CAG promoter-EGFP-pA* (pCriMGET-pCAG-EGFP-pA) and microinjected it into the pronuclei of pronuclear-stage mouse zygotes together with syn-crRNA-TS-crRNA, tracrRNA and Cas9 protein (Fig. 5A). After transplantation into pseudopregnant mice, E17.5 embryos were collected and subjected to genotyping PCR (Fig. 5B, Fig. S3A). In addition, by using the genomes from heterozygous and homozygous *H2B-EGFP* mice as controls, we determined the copy number of the integrated donor cassette in each embryo (Fig. S3B). We defined a transgenic fetus as one that carries more than one copy of the integrated donor transgene (*EGFP*). The results showed that 20.3% (13/64) of the embryos were transgenic (Fig. 5C). We also confirmed EGFP protein expression in the transgenic embryos (Fig. 5D). Notably, the integration efficiency was much lower in the absence of syn-crRNA-TS-crRNA (Fig. S4A–C), indicating that linearisation of the donor cassette with syn-crRNA-TS-crRNA enhances transgene integration. These results demonstrate that pCriMGET is useful for the efficient generation of transgenic mice.

### One-step generation of in-frame knock-in mice with pCriMGET

**Figure 6:**
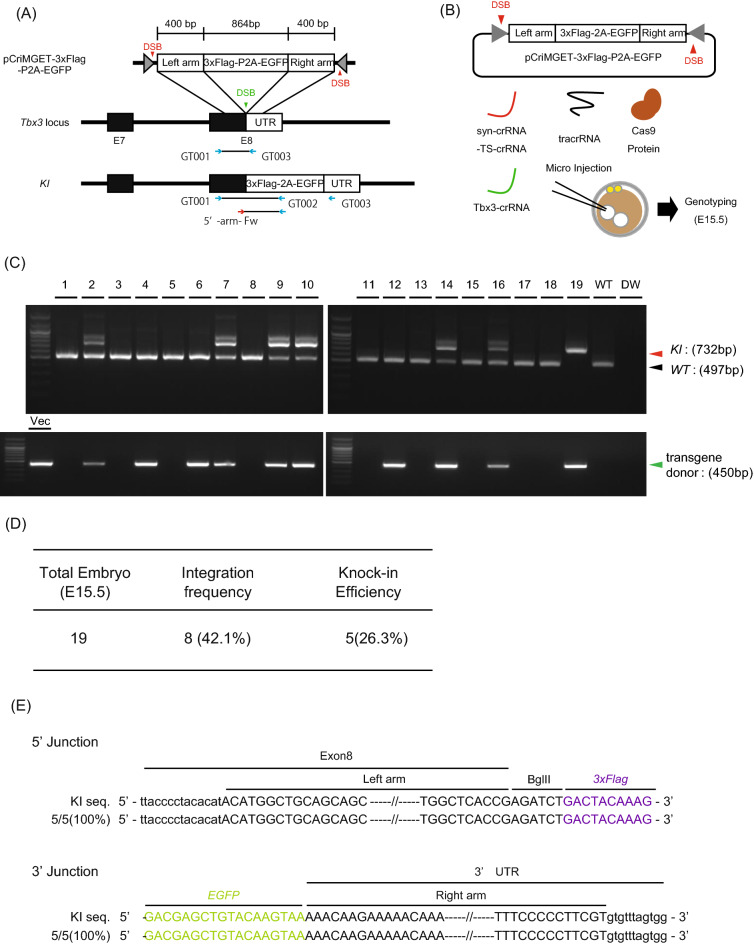
Generation of *Tbx3-3*×*Flag-P2A-EGFP* knock-in mice via pCriMGET system. (**A**) Schematic overview of pCriMGET-mediated in-frame knock-in (*KI*) strategy at the *Tbx3* gene locus. *3*×*Flag-P2A-EGFP* transgene was inserted into the 3′-end of the *Tbx3* coding sequence on exon 8 (E8), followed by the 3′ untranslated region (UTR). (**B**) Strategy for generating *Tbx3-3*×*Flag-P2A-EGFP* knock-in mice via the pCriMGET system. (**C**) Genotyping PCR for *Tbx3-3*×*Flag-P2A-EGFP* knock-in mouse embryos at E15.5. Upper panel shows *KI* (732 bp) and *WT* (497 bp) bands using PCR primer sets of GT001/GT002 and GT001/GT003 shown in (**A**), respectively. Lower panel shows transgene donor bands (450 bp) using the PCR primer set of 5′-arm-Fw/GT002 shown in (**A**). Vec: PCR product from pCriMGET-3×Flag-P2A-EGFP transgene cassette used as a positive control. (**D**) Integration frequency and knock-in efficiency of the embryos. (**E**) Sequence analysis of knock-in embryos. PCR products amplified from the 5′- and 3′-junction regions from each knock-in embryo were sequenced. Upper- and lower-case letters indicate sequences inside and outside of the donor cassette, respectively.

**Figure 7:**
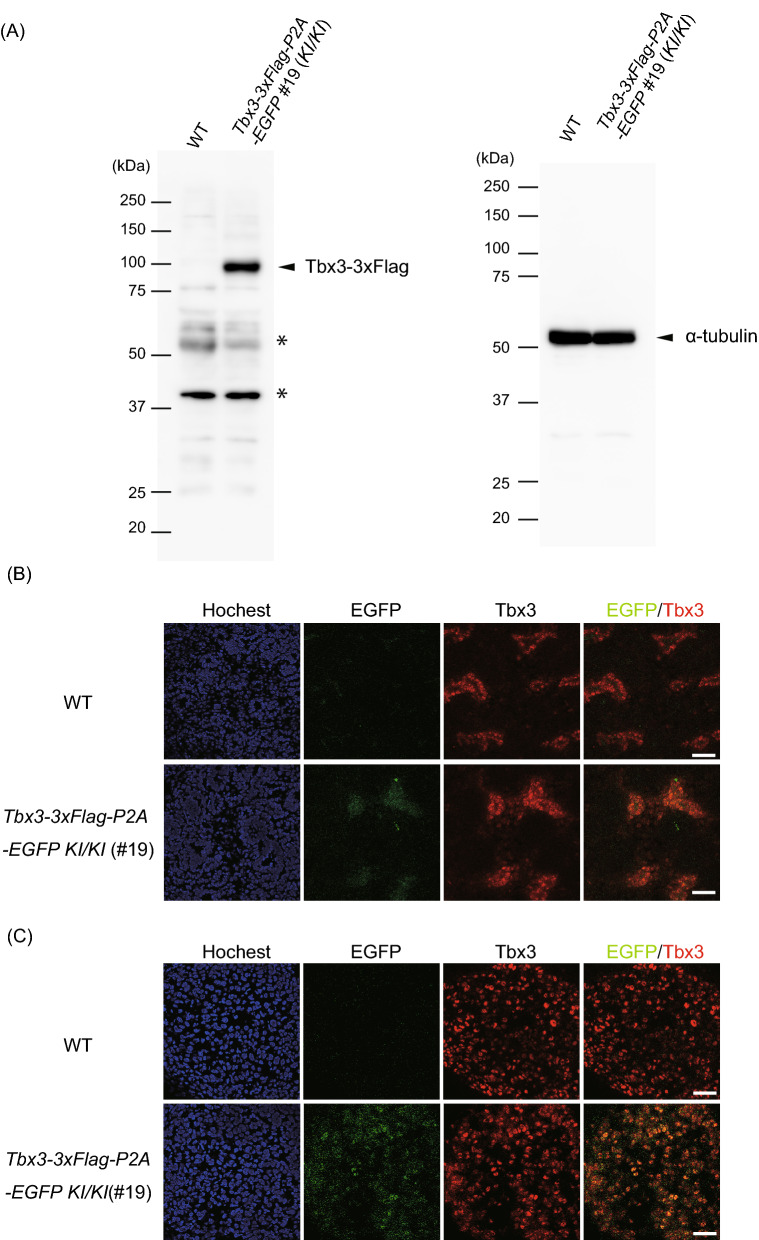
Donor transgene expression in *Tbx3-3*×*Flag-P2A-EGFP* knock-in mice. (**A**) Western blotting for Tbx3-3×Flag (left) and control α-tubulin (right) protein expression in E15.5 *WT* and *Tbx3-3*×*Flag-P2A-EGFP KI* embryos (#19 in Fig. 6). Asterisks show nonspecific bands. (**B**), (**C**) Representative immunofluorescence images of kidney (**B**) and adrenal gland (**C**) in *WT* and *Tbx3-3*×*Flag-P2A-EGFP KI* embryos. EGFP (green), Tbx3 (red) and Hoechst (blue). Scale bar, 20 μm.

Given the highly efficient integration of a transgene donor in mice, we further examined whether pCriMGET is also useful for the in-frame knock-in of exogenous DNA in mice. We designed a strategy for the in-frame integration of a donor gene encoding *3*×*Flag-P2A-EGFP* into the 3′-end of the *Tbx3*-coding sequence on exon 8 (Fig. 6A). We constructed pCriMGET-3×Flag-P2A-EGFP, which incorporates the donor gene encoding *3*×*Flag-P2A-EGFP* flanked by 400-bp homology arms, and microinjected it into the pronuclei of pronuclear-stage mouse embryos together with syn-crRNA-TS-crRNA, Tbx3-crRNA^24^, tracrRNA and Cas9 protein (Fig. 6B). After transplantation into pseudopregnant mice, E15.5 embryos were collected and subjected to genotyping PCR (Fig. 6C). We also determined the copy number of the transgene in each donor gene-integrated embryo (Fig. S5). The results showed that, out of a total of 19 embryos, 8 embryos (42.1%) were transgenic and 5 (26.3%) were knock-in with more than one copy of the transgene (Fig. 6D). We detected no indels or frame-shift in the 5′ and 3′ junction regions of all knock-in embryos (Fig. 6E). We confirmed the expression of Tbx3-3×Flag protein and EGFP protein in the E15.5 knock-in embryo (No. 19) by western blotting and immunohistochemistry, respectively (Fig. 7A–C). Additionally, we also obtained 4 knock-in pups out of 11 pups using the same strategy (Fig. S7A,B). Moreover, we confirmed that the transgene was inherited by F_1_ pups in a Mendelian manner (Fig. S7C,D). We also checked for off-target effects of syn-crRNA-TS-crRNA. We searched putative off-target regions in the mouse genome that matched more than 17 out of the 20 bases of syn-crRNA-TS using the free software Cas-OFFinder, and identified five candidate regions. We sequenced these genomic regions and found no indel mutations in all donor gene-integrated embryos (Table S1).

Notably, the knock-in efficiency of the pCriMGET method was comparable to that of the Tild-CRISPR method, in which in vitro cleaved linearised donor cassette was used as a repair template (Fig. S6A,B,D). In addition, the knock-in efficiency was much lower in the absence of syn-crRNA-TS-crRNA (Fig. S6A,C,D), indicating that linearisation of the pCriMGET donor cassette with syn-crRNA-TS-crRNA enhances knock-in efficiency.

Finally, to assess the versatility of the pCriMGET method for in-frame knock-in, we designed a strategy for the in-frame integration of a donor gene encoding *IRES-hDTR-2A-EGFP* into the *Clec4f* 3′UTR in the exon 7 genomic locus (Fig. S8A). We constructed pCriMGET-IRES-hDTR-2A-EGFP with 500-bp homology arms and microinjected it into the pronuclei of pronuclear-stage mouse embryos together with syn-crRNA-TS-crRNA, Clec4f-crRNA^25^, tracrRNA and Cas9 protein (Fig. S8B). After transplantation into pseudopregnant mice, 4-week-old male and female mice were collected and subjected to genotyping PCR (Fig. S8C). Out of a total of 17 pups, 2 pups (11.8%) were knock-in (Fig. S8D) with no indels or frame-shift in the 5′ and 3′ junction regions, implying the versatility of the pCriMGET method in generating knock-in mice. These results taken together demonstrate that pCriMGET is useful for the precise and efficient generation of in-frame knock-in mice.

## Discussion

In this study, we developed pCriMGET, a CRISPR/Cas9-cleavable donor plasmid equipped with syn-crRNA-TS of minimal off-target potential. Compared with the previously described HMEJ-based method, where crRNA on-target sequences sandwich a donor cassette^18,19^, pCriMGET is simple in construction because the donor cassette can be incorporated into MCS flanked by the syn-crRNA-TSs. Similar to the HMEJ-based and Tild-CRISPR method, the pCriMGET system achieved much higher targeted knock-in efficiency in mouse embryos than the conventional HR-based method^4,5^. Moreover, unlike the Easi-CRISPR method^14,16^, the pCriMGET system appears not to be particularly limited in terms of the transgene donor size: 3.0–3.3 kb of exogenous DNA (*EF1α-hygro-T2A-EGFP-pA* and *Clec4f-hDTR-2A-EGFP*) was successfully integrated into the genome.

The pCriMGET system accomplished the efficient generation of not only knock-in mice, but also transgenic mice. It has been reported that a linearised donor cassette is more efficiently integrated into genomic DNA than a circular plasmid^26,27^. In the pCriMGET system, the donor cassette is linearised in vivo by CRISPR/Cas9 via syn-crRNA-TS, enabling the frequency of transgene insertion into the genome to be increased. The efficiency of generation of transgenic mice by the pCriMGET system (> 20%) was comparable to that of the conventional linearised-donor cassette injection method (20–30%)^26^, highlighting its easy-to-manipulate linearised-donor gene preparation.

The pCriMGET system exhibited nontargeted donor gene integration with various copy numbers (Fig. 6D, Fig. S5). Previous studies showed that the frequency of nontargeted donor gene integration correlated with the length of the homology arms^17–19^. In HMEJ-based and Tild-CRISPR methods, the optimised minimum length of homology arms was 800–900 bp. The homology arms used in this study were 400 bp in length, which would cause nontargeted donor integration. However, the pCriMGET system achieved 26.3% in-frame knock-in embryos, even using relatively short homology arms. In addition, *Tbx3-3*×*Flag-P2A-EGFPKI* mice generated by the pCriMGET system exhibited transgene germline transmission in accordance with Mendel’s law, implying that backcrossing the mutant mice to the inbred control strain would eliminate the randomly integrated donor cassettes. Taking these findings together, the pCriMGET system is a simple, versatile, low-cost and efficient genome editing tool for the generation of transgenic and gene-targeted mice.

The remaining issues of the pCriMGET system include mosaicism, a commonly raised problem when generating mutant animals by using CRISPR/Cas9^28–31^. Our results showed that several mutant embryos generated by the pCriMGET system exhibited mosaicism with less than one copy of the donor gene (Fig. S3, S5). Another remaining issue is regional variability of knock-in efficiency; *Clec4f-hDTR-2A-EGFPKI* mice were generated at low efficiency compared with *Tbx3-3*×*Flag-P2A-EGFPKI* mice (Fig. 6, Fig. S7, S8). It has been suggested that nucleosome-assembled genomic regions are protected from Cas9-mediated cleavage^32–36^, implying that genomic structures influence the efficiency of pCriMGET-mediated genome editing. These issues should be addressed in future studies.

## Methods

### pCriMGET

The crRNA-TSs in SPA of the rabbit β-globin gene and putative off-target regions of each crRNA-TS in mouse (GRCm38/m10) and human (GRCh38/hg38) genomes were searched using the free software CRISPRdirect. The SPA sequence including syn-crRNA-TS was amplified by PCR using the single-stranded DNA (5′-GTTTTTTGTGTGAATCGATAGTACTAACATACGCTCTCCATCAAAACAAAACGAAACAAAACAAACTAGCAAAATAGGCT-3′) as a template. To reduce CRISPR-Cas9 off-target effects, three nucleotide mutations (see Fig. S1) were introduced in SPA by reverse primers. KpnI/XhoI sites and BamHI/XbaI sites were introduced into the 5′-modified (m)SPA and 3′-mSPA, respectively, by PCR primers. Then, the 5′-mSPA and 3′-mSPA were inserted into KpnI-XhoI and BamHI-XbaI sites on the pBluescriptII SK(+) plasmid, respectively. We validated the insertion by sequencing.

### pCriMGET-EF1α-hygro-T2A-EGFP-pA

The hygromycin resistance gene was amplified using the hygromycin selection cassette (GENE BRIDGES: A011) as a template and inserted into KpnI-BamHI sites on the pcDNA3-EF1α plasmid. T2A peptide-coding sequence (5′-GAGGGCAGAGGAAGTCTTCTAACATGCGGTGACGTGGAGGAGAATCCCGGCCCT-3′) was fused to the 5′-end of the EGFP coding sequence, which was PCR-amplified from pEGFP-C1 (Clontech). Then, the T2A-EGFP coding sequence was inserted into NheI-XbaI sites on the pcDNA3-EF1α-hygro plasmid. The EF1α promoter-hygro-T2A-EGFP-bGHpA coding sequence was amplified by PCR and inserted into the SalI site of pCriMGET MCS.

### pCriMGET-resT2A-mCherry-stop

sgRNA resistant T2A (resT2A) sequence (5′-GAGGGCAGAGGAAGTCTTCTAACATGCGGTGACGTGGAAGAAAACCCTGGACCT-3′) and 3× stop codons were fused to the 5′-end and 3′-end of the mCherry coding sequence, respectively, by PCR amplification using pcDNA3-EF1 α-mCherry as a template. The resT2A-mCherry-stop coding sequence was inserted into NheI-BamHI sites of pcDNA3-EF1α-hygro-T2A-EGFP. Then, the resT2A-mCherry-stop coding sequence with homology arms of various lengths was PCR-amplified using pcDNA3-EF1α-hygro-resT2A-mCherry-stop-EGFP as a template and inserted into HidIII-EcoRI sites of pCriMGET MCS.

### pCriMGET-SA-neo-pA

The human genomic sequences for 400 bp upstream and downstream of the AAVS1 site within the *PPP1R12C* gene locus were PCR-amplified using HEK293T genomic DNA as a template and used as homology arms. The splicing acceptor (SA) sequence (5′-CTTCTGACCTCTTCTCTTCCTCCCACAGG-3′) was fused to the 5′-end of T2A-EGFP cDNA by PCR amplification. The SA-T2A-EGFP coding sequence and the homology arms were fused and inserted into SalI-EcoRI sites of pCriMGET MCS by using NEBuilder HiFi DNA Assembly Master Mix (New England Biolabs), in accordance with the manufacturer’s protocol. Then, the EGFP gene of pCriMGET-SA-T2A-EGFP was replaced by a neomycin resistance gene using NEBuilder HiFi DNA Assembly Master Mix.

### pCriMGET-pCAG-EGFP-pA

EGFP cDNA was PCR-amplified from pEGFP-C1 and inserted into XbaI-XhoI sites of pCAG-EGxxFP^37^ (Addgene #50716). Then, the CAG promoter-EGFP-rabbit-pA coding sequence was PCR-amplified from pCAG-EGFP and inserted into HindIII-EcoRI sites of pCriMGET MCS.

### pCriMGET-3×Flag-P2A-EGFP

The mouse genomic sequences for 400 bp upstream and downstream of the stop codon of the *Tbx3* gene were PCR-amplified using C57BL/6JJcl mouse genomic DNA as a template, and were used as left and right homology arms, respectively. The 3×Flag-tag coding sequence was fused to the 3′-end of the left homology arm by PCR. P2A coding sequence (5′-GGCTCGGGTGCCACGAATTTCTCATTACTGAAGCAGGCTGGAGACGTGGAGGAGAACCCTGGACCT-3′) was fused to the 5′-end of the EGFP gene by PCR. Then, the fragments of the left arm-3×Flag, P2A-EGFP, and the right arm were fused and inserted into the EcoRV site of pCriMGET MCS using NEBuilder HiFi DNA Assembly Master Mix (New England Biolabs).

### pCriMGET-Clec4f-IRES-hDTR-P2A-EGFP

The mouse genomic sequences for 500 bp upstream and downstream of the crRNA target site of the *Clec4f* gene were PCR-amplified using C57BL/6JJcl mouse genomic DNA as a template, and were used as left and right homology arms, respectively. Human diphtheria toxin receptor (hDTR) carrying I117V/L148V mutations was constructed using three fragments amplified from HEK293T genomic DNA by PCR, and these three fragments were fused and inserted into the EcoRI-BamHI site of pEGFP-N3 (Clontech) using NEBuilder HiFi DNA Assembly Master Mix (New England Biolabs). The internal ribosome entry site (IRES) was amplified from pQCXIN X2/pTER shLUC (w347-1) (Addgene: #17489), hDTR carrying I117V/L148V was amplified from pEGFP-N3-hDTR, and P2A-EGFP was amplified from pCriMGET-Tbx3-3xFlag-P2A-EGFP plasmid by PCR. Then, the fragments of the left arm, IRES, hDTR carrying I117V/L148V, P2A-EGFP, and the right arm were fused and inserted into the XhoI-EcoRI site of pCriMGET MCS using NEBuilder HiFi DNA Assembly Master Mix (New England Biolabs).

### pX330-syn-crRNA-TS-sgRNA, pX330-T2A-sgRNA and pX330-AAVS1-sgRNA

The syn-crRNA-TS-sgRNA and T2A-sgRNA sequences were designed using free software (CRISPRdirect). The AAVS1-sgRNA sequence was as previously described^22^. The DNA sequences coding each sgRNA used in this study are listed in Table S2. The oligonucleotides encoding each sgRNA were annealed and ligated into the BbsI site of pX330 (Addgene #42230).

### Cell culture and transfections

HeLa cells and HEK293T cells (RIKEN BRC, RCB2202) were cultured in DMEM (Nissui) containing 10% foetal bovine serum (GIBCO), 4 mM-L-glutamine (Nacalai) and 0.2% sodium bicarbonate. HeLa cells and HEK293T cells were transfected with plasmids using PEI-MAX (Polysciences) and Lipofectamine3000 (Invitrogen), respectively, in accordance with the manufacturers’ protocols.

### Genomic insertion of donor genes in culture cells

HEK293T cells were transfected with pCriMGET-EF1α-hygro-T2A-EGFP-pA and pX330-syn-crRNA-TS-sgRNA. The transfection efficiency was analysed by flow cytometry 24 h after transfection. Cells were seeded on a 35-mm dish (10^4^ cells per well) and selected using hygromycin (250 µg/mL) for 14 days. Surviving colonies were stained by crystal violet. Colony formation efficiency was calculated as follows: (colony number/10^4^ cells × transfection efficiency) × 100 (%).

### Hygro-T2A-EGFP reporter cell line

HEK293T cells were transfected with pcDNA3-EF1 α-hygro-T2A-EGFP and stably transduced cells were selected using hygromycin (250 µg/mL). After 14 days of antibiotic selection, single colonies were isolated and subjected to flow cytometry to examine EGFP expression. We established three reporter cell lines and used one cell line for the analysis.

### Flow cytometry

Cells were dissociated with PBS containing 0.25% trypsin and 1 mM EDTA (Nacalai) and analysed on a BD LSRFortessa X-20. The data were analysed using FlowJo software (BD).

### Donor gene knock-in into *AAVS1* locus in culture cells

HeLa cells were transfected with plasmids of pCriMGET-SA-neo-pA, pX330-syn-crRNA-TS-sgRNA and pX330-AAVS1-sgRNA. After 24 h, cells were seeded on a 35-mm dish (5.0 × 10^3^ cells per well) and selected by G418 (700 µg/mL) for 14 days. Surviving colonies were stained by crystal violet and knock-in efficiency was calculated as follows: (colony numbers/5.0 × 10^3^ cells) × 100 (%). The transfection efficiency for each sample was analysed 24 h after transfection by PCR amplification of the *ampicillin resistance gene* (*Amp*) coded on pCriMGET and pX330.

### crRNA, tracrRNA and Cas9 protein

Syn-crRNA-TS-crRNA (5′-GCUGUCCCCAGUGCAUAUUCguuuuagagcuaugcuguuuug-3′), Tbx3-crRNA (5′-AGCUUUAGUUCUUGGUGCGCguuuuagagcuaugcuguuuug-3′)^38^ and tracrRNA (5′-aaacagcauagcaaguuaaaauaaggcuaguccguuaucaacuugaaaaaguggcaccgagucggugcu-3′)^27^ were obtained from FASMAC. Syn-crRNA-TS-crRNA (5′-GCUGUCCCCAGUGCAUAUUCguuuuagagcuaugcu-3′), Clec4f-crRNA (5′-ACGACAGGGCAAUACAGGACguuuuagagcuaugcu-3′) and Alt-R CRISPR-Cas9 tracrRNA (Integrated DNA Technologies, IDT #1072532) were obtained from IDT. Recombinant *Streptococcus pyogenes* Cas9 protein was purchased from Invitrogen (TureCut Cas9 Protein v2: A36497).

### Pronuclear injection

C57BL/6J female mice were superovulated and oocytes were in vitro fertilised with C57BL/6J sperm. Fertilised eggs were cryopreserved by vitrification^39^ at the pronuclear stage. One hour after thawing, morphologically normal zygotes were cultured in KSOM medium (ARK Resource) and microinjected. For the generation of *EGFP* transgenic mice, pCriMGET-pCAG-EGFP-pA (25 ng/µL), syn-crRNA-TS-crRNA (50 ng/µL), tracrRNA (100 ng/µL) and Cas9 protein (100 ng/µL) were microinjected into the pronuclei of zygotes. For the generation of *Tbx3-flag-EGFP* knock-in mice, pCriMGET-3×Flag-P2A-EGFP (25 ng/µL), Tbx3-crRNA (50 ng/µL), syn-crRNA-TS-crRNA (10 ng/µL), tracrRNA (100 ng/µL) and Cas9 protein (100 ng/µL) were microinjected into the pronuclei of zygotes. For the generation of *Clec4f-IRES-hDTR-P2A-EGFP* knock-in mice, pCriMGET-IRES-hDTR-P2A-EGFP (25 ng/µL), Clec4f-crRNA (50 ng/µL), syn-crRNA-TS-crRNA (10 ng/µL), tracrRNA (100 ng/µL) and Cas9 protein (100 ng/µL) were microinjected into the pronuclei of zygotes. The microinjection was performed using a Leica Micromanipulator and Eppendorf CellTram Vario. The injected zygotes were cultivated in KSOM medium overnight and then transferred into the oviducts of pseudopregnant ICR mice.

### Genotyping PCR

For *EGFP* transgenic mice, genomic DNA extracted from E17.5 embryo tails was amplified by PCR with KOD Plus Neo (TOYOBO) using primer sets designed inside of the donor cassette. PCR amplification of the genomic region of the potential syn-crRNA-TS off-target sequence on chromosome 8 (see Table S1, CriMGET-OT1) was used as an internal control. *Rosa26-lox-stop-lox-H2B-EGFP*^40^ (R26R-H2B-EGFP; RIKEN CDB0203K) heterozygous mouse tail genome was used as a reference. For *Tbx3-3xFlag-2A-EGFP* knock-in mice, genomic DNA extracted from E15.5 embryo tails was amplified by PCR using primer sets designed inside and outside of the donor cassette. For *Clec4f-IRES-hDTR-P2A-EGFP* knock-in mice, genomic DNA extracted from 4-week-old male and female mouse tails was amplified by PCR using primer sets designed inside and outside of the donor cassette. The primers used for the genotyping PCR are listed in Table S3.

### Genotyping PCR for blastocysts

For *EGFP* transgenic and *Tbx3-3xFlag-2A-EGFP* knock-in lines, each blastocyst was put into a PCR tube containing 10 µL of lysis buffer [2 mM Tris–Cl (pH8.0), 0.5 mM EDTA, 40 mM NaCl, 0.1% Tween 20, 0.1% Triton-X 100, and 4 μg/mL Proteinase K] using a glass capillary under a dissection microscope. The samples were incubated for 30 min at 56 °C, followed by incubation for 10 min at 95 °C to inactivate proteinase K. PCR was carried out with 2 µL lysis samples using KOD One PCR Master Mix (TOYOBO).

### Copy number analysis of transgene donor cassette

For determination of the copy number of the transgene donor cassette in *EGFP* transgenic mice and *Tbx3-3xFlag-2A-EGFP* knock-in mice, real-time PCR was performed in the E17.5 and E15.5 embryonic tail genomes. qPCR was conducted in duplicate with 20 ng of DNA in a 20 µL reaction mixture using the THUNDERBIRD SYBR qPCR Mix (TOYOBO) and the Applied Biosystems 7500 Real-Time system (Applied Biosystems). *R26R-H2B-EGFP* heterozygous and homozygous knock-in mouse tail genomes were used as references for copy number. DNA amount was calibrated using the PCR product of the CriMGET-OT1 region on chromosome 8 as an internal control. Copy number was defined as the value relative to the *R26R-H2B-EGFP* heterozygous knock-in sample.

### Western blot

*Wild-type* and *Tbx3-flag-EGFP* knock-in E15.5 embryos were lysed in RIPA buffer [50 mM Tris–Cl (pH 7.6), 150 mM NaCl, 1% NP-40, 0.5% sodium deoxycholate, 0.1% SDS, 10 µg/mL aprotinin, 10 µg/mL leupeptin and 1 mM PMSF] and centrifuged at 15,000 rpm and 4 °C for 15 min. The supernatants were subjected to western blotting using anti-FLAG (mouse, F3165; Sigma-Aldrich) and anti-α-tubulin (mouse, T6199; Sigma-Aldrich) primary antibodies, peroxidase-conjugated secondary antibodies (GE Healthcare) and Western Lighting ECL reagents (Perkin Elmer), in accordance with the manufacturers’ instructions. Unprocessed full-length membrane blots are shown in Fig. S9.

### Immunohistochemistry

*EGFP* transgenic E17.5 embryos and *Tbx3-flag-EGFP* knock-in E15.5 embryos were cryoprotected in 20% sucrose/PBS and frozen in optimal cutting temperature compound. The samples were sectioned and subjected to immunostaining. The tissue samples were fixed with 4% paraformaldehyde, followed by permeabilisation with 0.5% Triton X-100 in TBS for 15 min at room temperature. Next, the sections were blocked with 5% bovine serum albumin at room temperature for 1 h, incubated with primary antibodies at 4 °C overnight, washed and then incubated for 1 h with secondary antibodies [Alexa Fluor 488-conjugated anti-chicken or Cy3-conjugated anti-rabbit (Jackson ImmunoResearch, West Grove, PA, USA)]. The following primary antibodies were used: anti-GFP (chicken, 1:2000, ab13970; Abcam) and anti-Tbx3 (rabbit, 1:200, ab99302; Abcam). The images of *EGFP* transgenic and *Tbx3-flag-EGFP* knock-in embryos were acquired using a Leica AF7000 fluorescence microscope and a Leica SP8 confocal microscope, respectively.

### Off-target analysis

A search for potential off-target sites in the mouse genome (GRCm38/mm10) was performed using Cas-OFFinder (https://www.rgenome.net/cas-offinder). Genomic regions that matched more than 17 out of 20 bases of the syn-crRNA-TS were PCR-amplified and sequenced to evaluate the off-target effects. The primers used in the analysis are listed in Table S3.

### Animals

C57BL/6JJmsSLC and Jcl:ICR mice were obtained from Japan SLC Inc. and CLEA Japan Inc., respectively. All experiments were performed in accordance with Kyoto University’s Regulations on Animal Experimentation. All animal experiments in this study were approved by the Committee for Animal Experiments of the Institute for Frontier Life and Medical Sciences, Kyoto University.

## Supplementary information

Supplementary Information.
