# Supplementary material for: Genome editing with the donor plasmid equipped with synthetic crRNA-target sequence
Source: Sci Rep. 2020 Aug 24;10:14120. doi: 10.1038/s41598-020-70804-6 (PMC7445171; doi:10.1038/s41598-020-70804-6)
Supplement: Supplementary file 1 — Supplementary Information. [file 41598_2020_70804_MOESM1_ESM.pdf]

Supplementary information

Genome editing with the donor plasmid equipped with synthetic crRNA-target sequence

Riki Ishibashi[1,2]*, Kota Abe[1,2], Nanami Ido[1,2],
Satsuki Kitano[1], Hitoshi Miyachi[1] and Fumiko Toyoshima[1,2]*

1. Department of Biosystems Science, Institute for Frontier and Medical Sciences, Kyoto University, Sakyo-ku, Kyoto 606-8507, Japan
2. Department of Mammalian Regulatory Networks, Graduate School of Biostudies, Kyoto University, Sakyo-ku, Kyoto 606-8502, Japan

*Corresponding authors:
Fumiko Toyoshima
E-mail: ftoyoshi@infront.kyoto-u.ac.jp
Tel.: +81-75-751-4015
Fax: +81-75-751-4037

Riki Ishibashi
E-mail: rishibas@infront.kyoto-u.ac.jp
Tel.: +81-75-751-4016
Fax: +81-75-751-4037

This PDF file includes :
Supplementary Figure S1 to S9
Supplementary Table S1 to S3

(A)

(WT-SPA)

AATAAAATATCTTTATTTTCATTACATCTGTGTGTTGGTTTTTTGTGTGAATCGATAGTACTAAC
ATACGCTCTCCATCAAAACAAAACGAAACAAAACAAACTAGCAAAATAGGCTGTCCCCAGTGC
AAGTGCAGGTGCCAGAACATTTCTCT

(Modified-SPA)

AATAAAATATCTTTATTTTCATTACATCTGTGTGTTGGTTTTTTGTGTGAATCGATAGTACTAAC
ATACGCTCTCCATCAAAACAAAACGAAACAAAACAAACTAGCAAAATAGGCTGTCCCCAGTGC
ATATTCAGGTGCCAGAACATTTCTCT

Syn-crRNA-TS

(B)

	position		strand	putative Syn-crRNA-TS (5' → 3')	mouse genome (GRCm38/mm10)		human genome (GRCh38/hg38)	
	start	end			TTTT in 20 mer	hit_20mer + PAM	TTTT in 20 mer	hit_20mer + PAM
WT-SPA	16	38	+	TTTTCATTACATCTGTGTGTTGG	+	0	+	0
	75	97	-	CCATCAAAACAAAACGAAACAAA	+	0	+	2
	93	115	+	ACAAAACAAACTAGCAAAATAGG	-	0	-	2
	115	137	+	GCTGTCCCCAGTGCAAGTGCAGG	-	0	-	1
	120	142	-	CCCCAGTGCAAGTGCAGGTGCCA	-	0	-	1
	121	143	-	CCCAGTGCAAGTGCAGGTGCCAG	-	0	-	1
	122	144	-	CCAGTGCAAGTGCAGGTGCCAGA	-	0	-	1
Modified-SPA	115	137	+	GCTGTCCCCAGTGCATATTCAGG	-	0	-	0
	120	142	-	CCCCAGTGCATATTCAGGTGCCA	-	0	-	0
	121	143	-	CCCAGTGCATATTCAGGTGCCAG	-	0	-	0
	122	144	-	CCAGTGCATATTCAGGTGCCAGA	-	0	-	0

Supplementary Figure S1. Information of WT- and modified-SPA.

(A) Sequences of the WT- and modified-SPA of the rabbit β-globin gene. The modified nucleotides in SPA and PAM sequence are shown in red and blue, respectively. The syn-crRNA-TS is underlined. (B) List of putative crRNA-TSs within the WT- and modified-SPA. T-rich sequence and the number of matched sequence in the mouse (GRCm38/m10) and human (GRCh38/hg38) genomes are shown on the right.

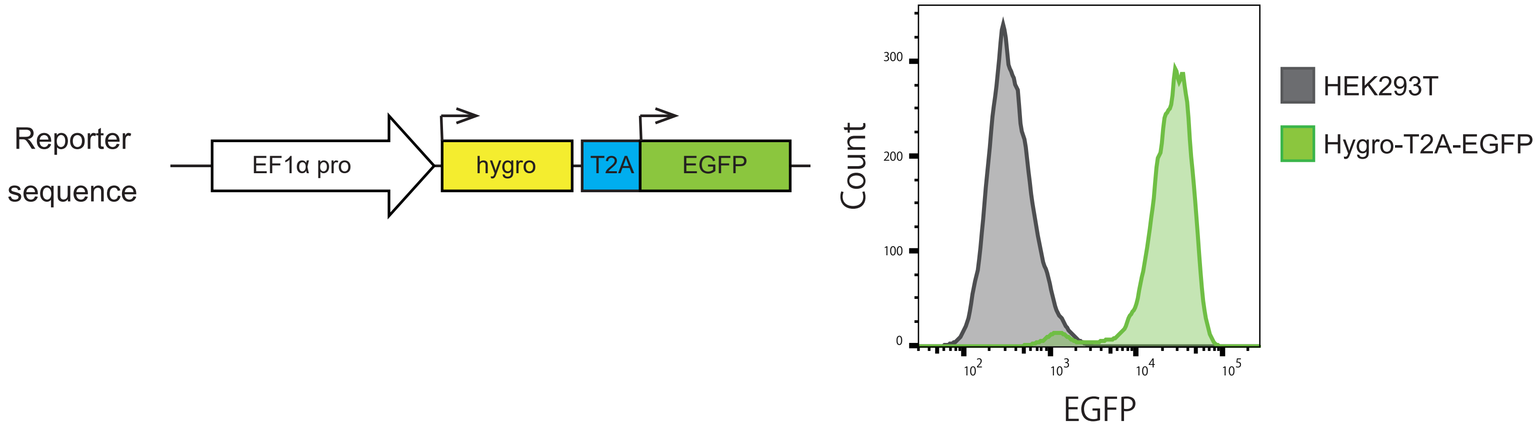

Supplementary Figure S2. *Hygro-T2A-EGFP* reporter HEK293T cell line.

Schematic of *EF1α-hygro-T2A-EGFP* reporter sequence (left). FACS analyses of EGFP expression in *WT*- and *hygro-T2A-EGFP* reporter HEK293T cells.

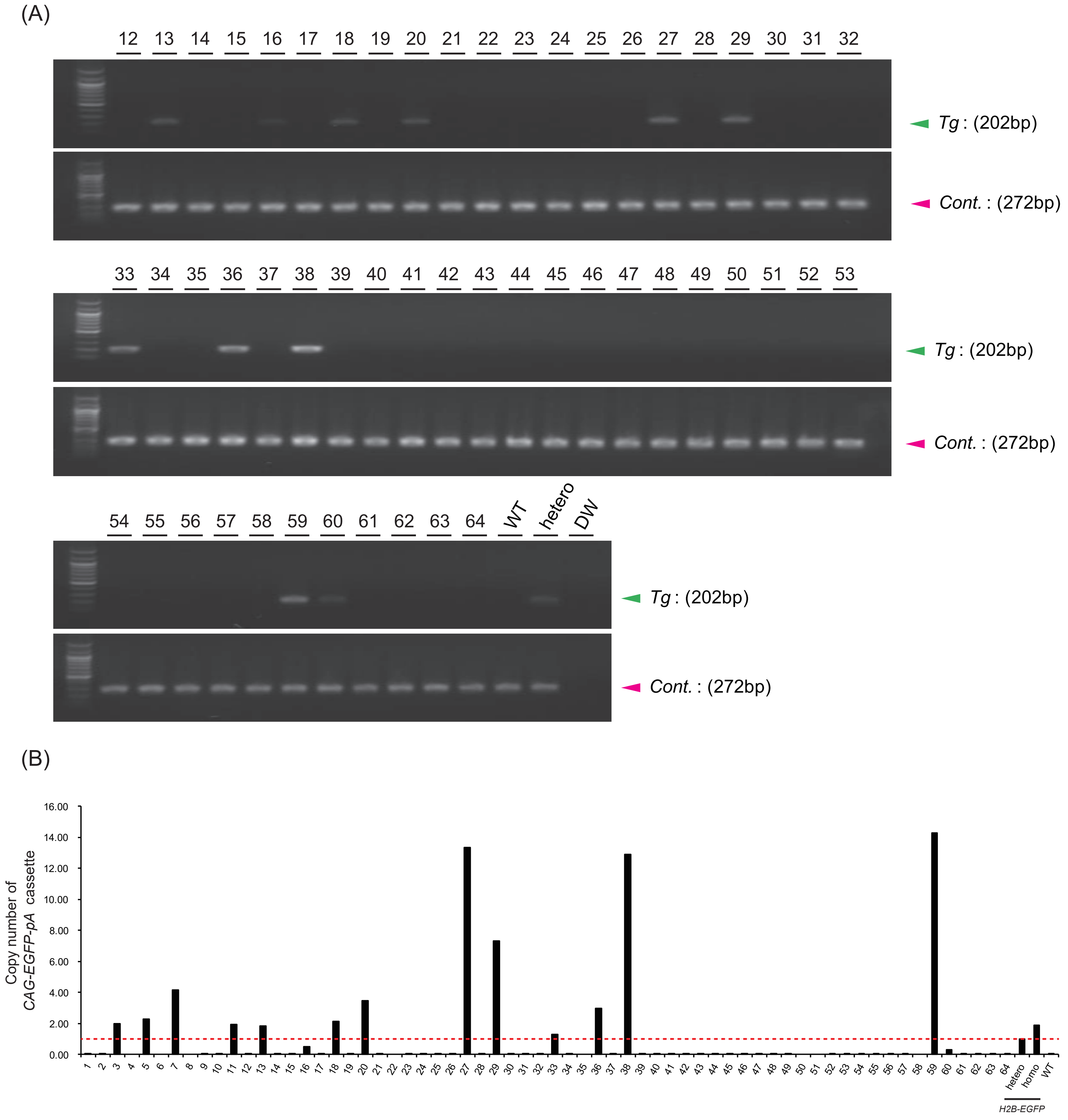

Supplementary Figure S3. Genotyping and donor gene copy number analysis of *CAG-EGFP* transgenic mice. (A) Genotyping PCR for *CAG-EGFP* transgenic mouse embryos at E17.5. Upper: PCR primer sets on the *pCAG-EGFP-pA* cassette yield a 202-bp band. Lower: PCR primer sets on the internal control genomic region on chromosome 8 yield a 272-bp band. The *WT* and *Rosa26-lox-stop-lox-H2B-GFP* heterozygous mouse (*hetero*) genomes were used as controls. DW: distilled water. (B) Copy number analysis of the *CAG-EGFP-pA* donor cassette in the genome of each embryo. *R26R-H2B-EGFP* heterozygous (*hetero*) and homozygous (*homo*) knock-in mouse tail genomes were used as references for copy number. Red dashed line shows the reference copy number of *EGFP* in the heterozygous mouse genome.

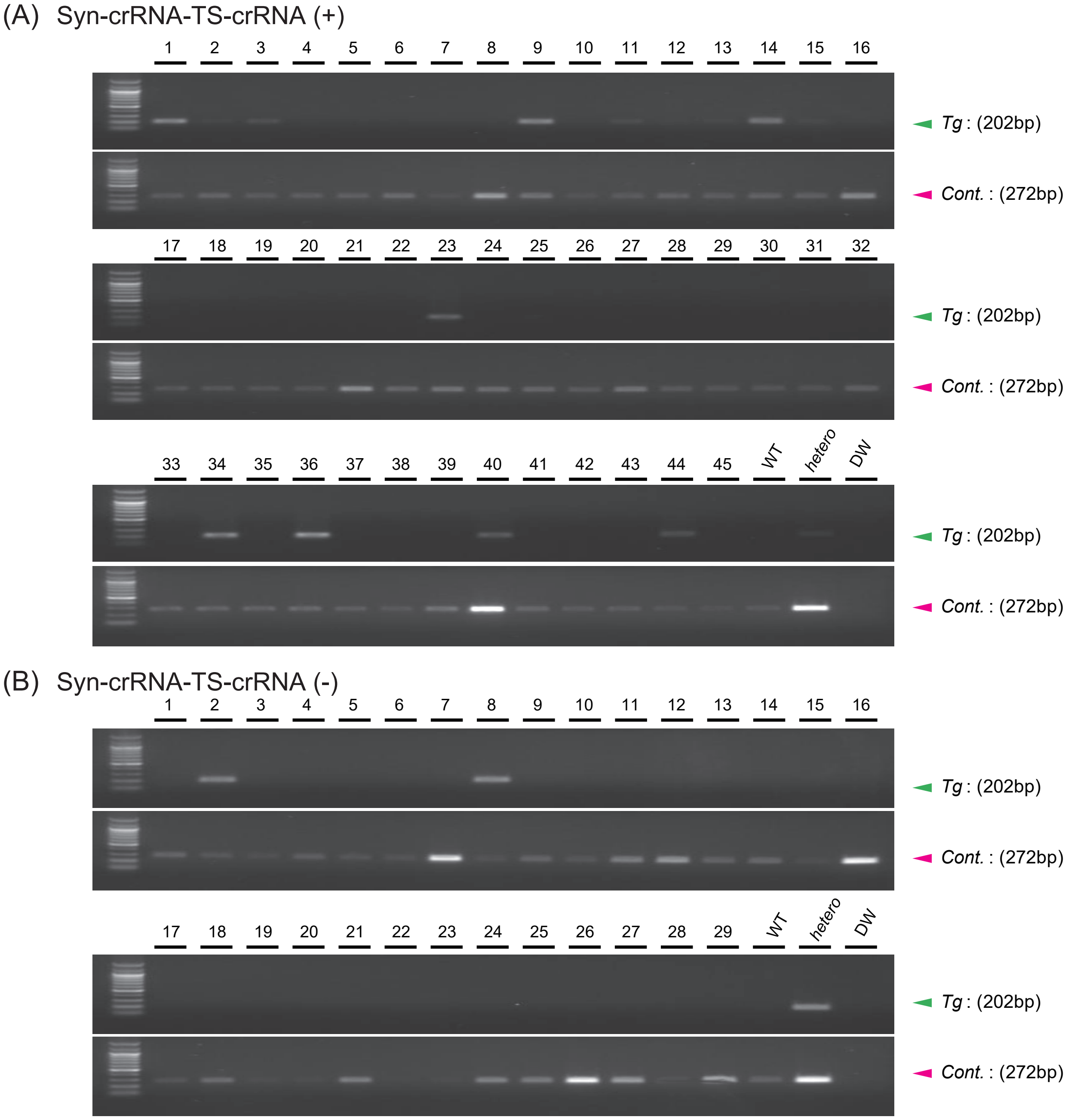

(C)

	Total Embryo (Blastocyst)	Integration frequency
Syn-crRNA -TS-crRNA (+)	45	10 (22.2%)
Syn-crRNA -TS-crRNA (-)	29	2 (6.9%)

Supplementary Figure S4. Generation of CAG-EGFP transgenic mice via pCriMGET with or without syn-crRNA-TS-crRNA.

(A) (B) Genotyping PCR for *CAG-EGFP* transgenic mouse blastocysts injected with pCriMGET-pCAG-EGFP-pA, tracrRNA and Cas9 protein together with (A) or without (B) syn-crRNA-TS-crRNA. Upper: PCR primer sets on the *pCAG-EGFP-pA* cassette yield a 202-bp band. Lower: PCR primer sets on the internal control genomic region on chromosome 8 yield a 272-bp band. The *WT* and *Rosa26-lox-stop-lox-H2B-GFP* heterozygous mouse (*hetero*) genomes were used as controls. DW: distilled water. (C) Integration frequency of the blastocysts.

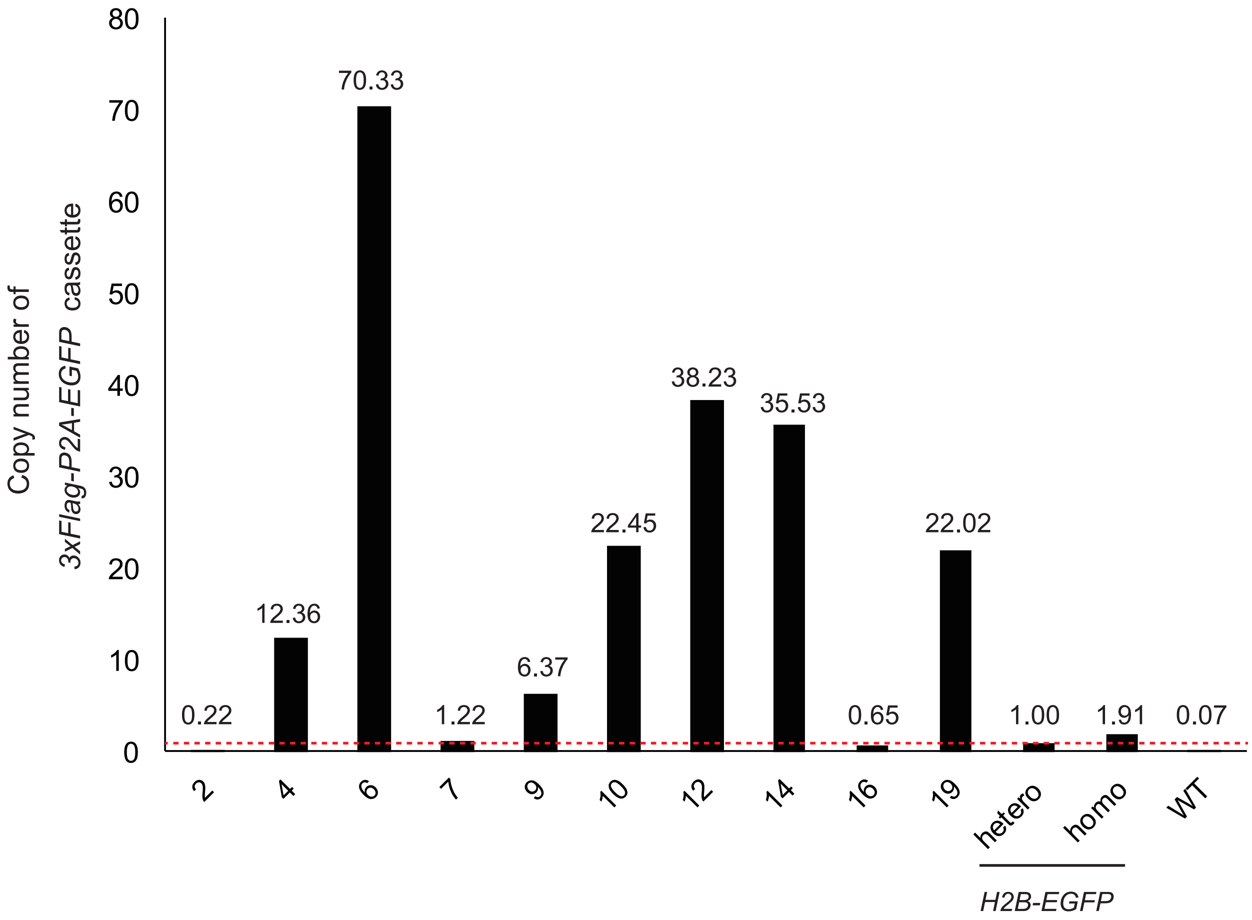

Supplementary Figure S5. Donor gene copy number analysis in *Tbx3-3xFlag-P2A-EGFP* knock-in mice.
Copy number analysis of the *3xFlag-P2A-EGFP* donor cassette in the genome of each donor gene-integrated embryo. *R26R-H2B-EGFP* heterozygous (*hetero*) and homozygous (*homo*) knock-in mouse tail genomes were used as references for copy number. Red dashed line shows the reference copy number of *EGFP* in the heterozygous mouse genome.

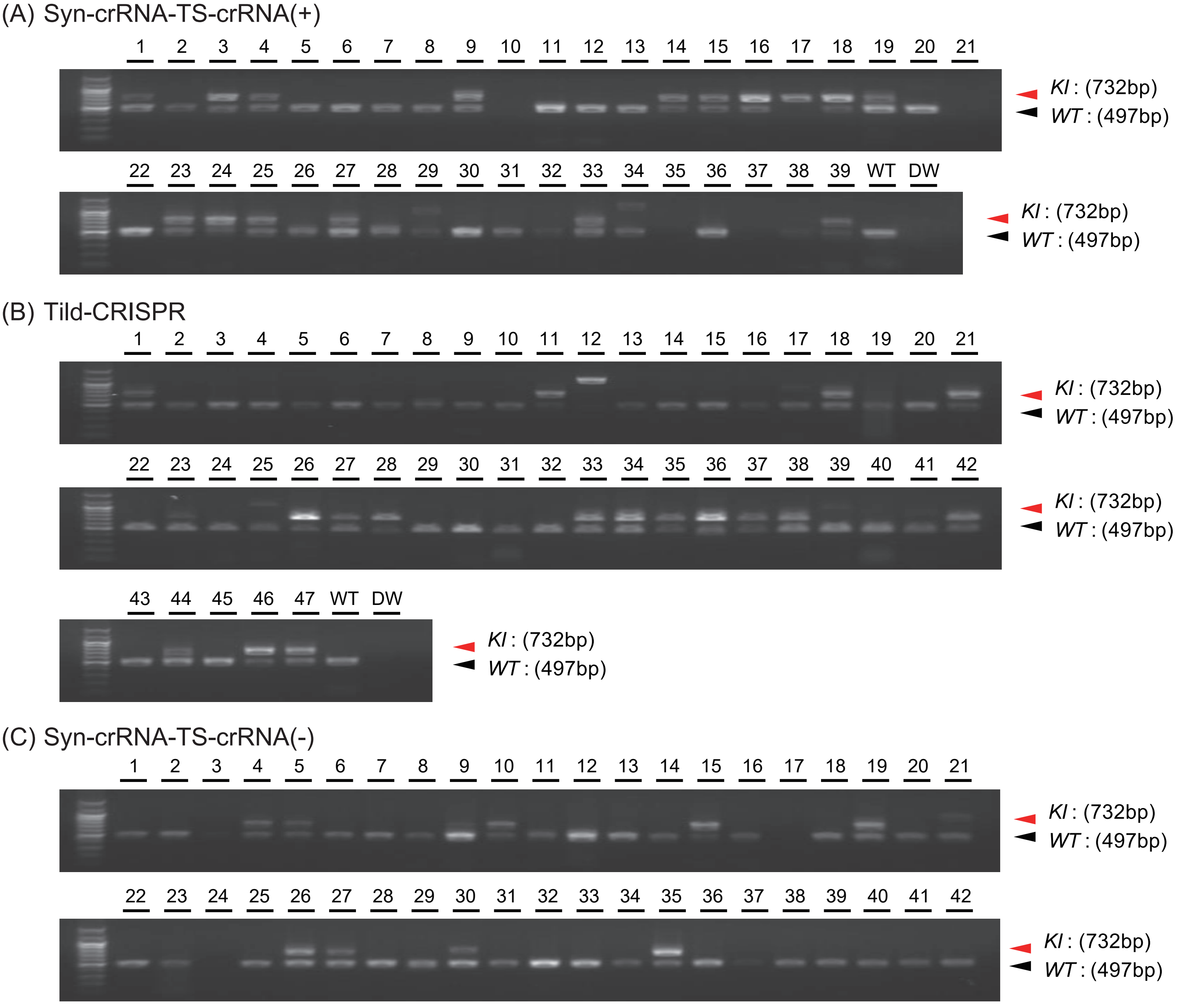

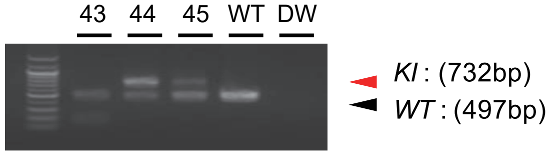

(D)

Knock-in frequency

Syn-crRNA-TS-crRNA(+)	Tild-CRISPR	Syn-crRNA-TS-crRNA(-)
16/35<br>(45.7%)	18/47<br>(38.3%)	12/43<br>(27.9%)

Supplementary Figure S6. Generation of *Tbx3-3xFlag-P2A-EGFP* knock-in mice via Tild-CRISPR vs pCriMGET with or without syn-crRNA-TS-crRNA.

(A) (B) (C) Genotyping PCR for *Tbx3-3×Flag-P2A-EGFP* knock-in mouse blastocysts generated via pCriMGET system (A), Tild-CRISPR system (B), and pCriMGET system without syn-crRNA-TS-crRNA (C). *KI* (732 bp) and *WT* (497 bp) bands used PCR primer sets of GT001/GT002 and GT001/GT003, respectively. (D) Knock-in frequency in blastocysts.

(A) *Tbx3-3xFlag-P2A-EGFP KI* (F0 : 4wk)

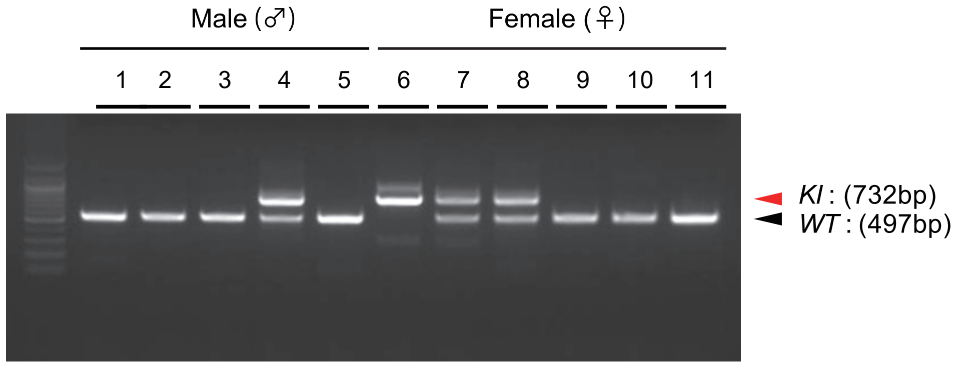

(B)

Total (4wk)	Knock-in Efficiency
11	4 (36.8%)

(C) *Tbx3-3xFlag-P2A-EGFP KI* (F1 : 4wk)

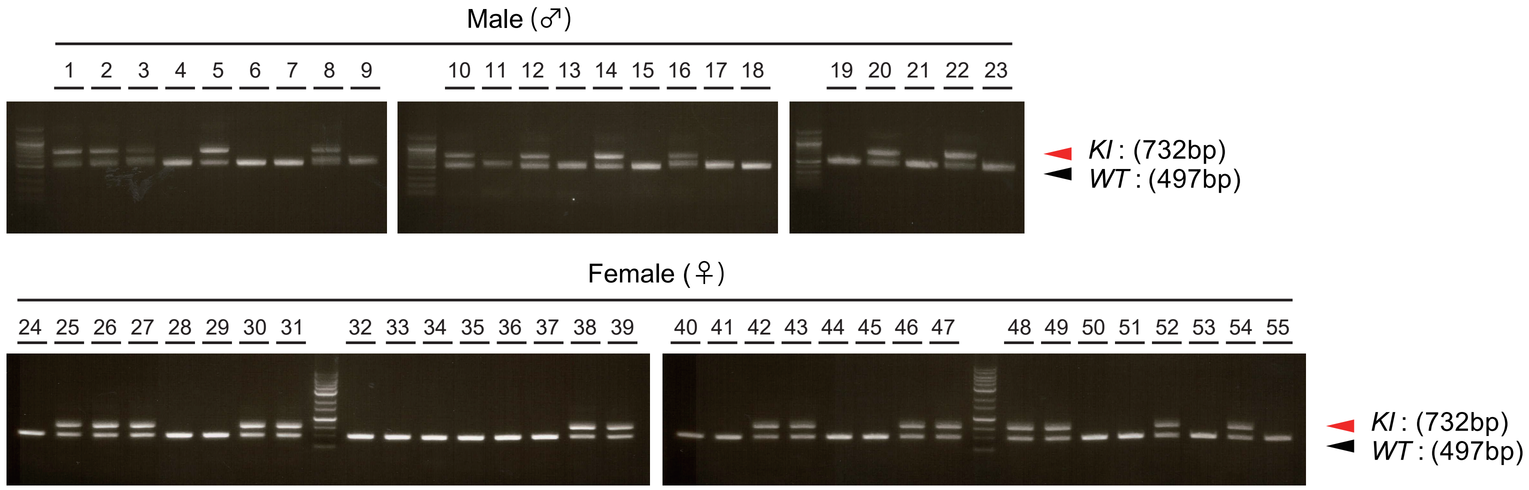

(D)

Total F1 pups (4wk)	Genotype frequency	
	WT	*KI*
55	29 (52.7%)	26 (47.3%)

Supplementary Figure S7. Germline transmission of the donor gene in Tbx3-3xFlag-P2A-EGFP knock-in mice. (A) Genotyping PCR for *Tbx3-3×Flag-P2A-EGFP* knock-in 4-week-old F0 pups. (B) Knock-in efficiency. (C) Genotyping PCR for 4-week-old F1 pups derived from the #4 *Tbx3-3×Flag-P2A-EGFP* knock-in male mouse which was mated with inbred control strain of female mice. (D) Genotype frequency of *WT* and *Tbx3-3×Flag-P2A-EGFP* knock-in alleles in 4-week-old F1 pups.

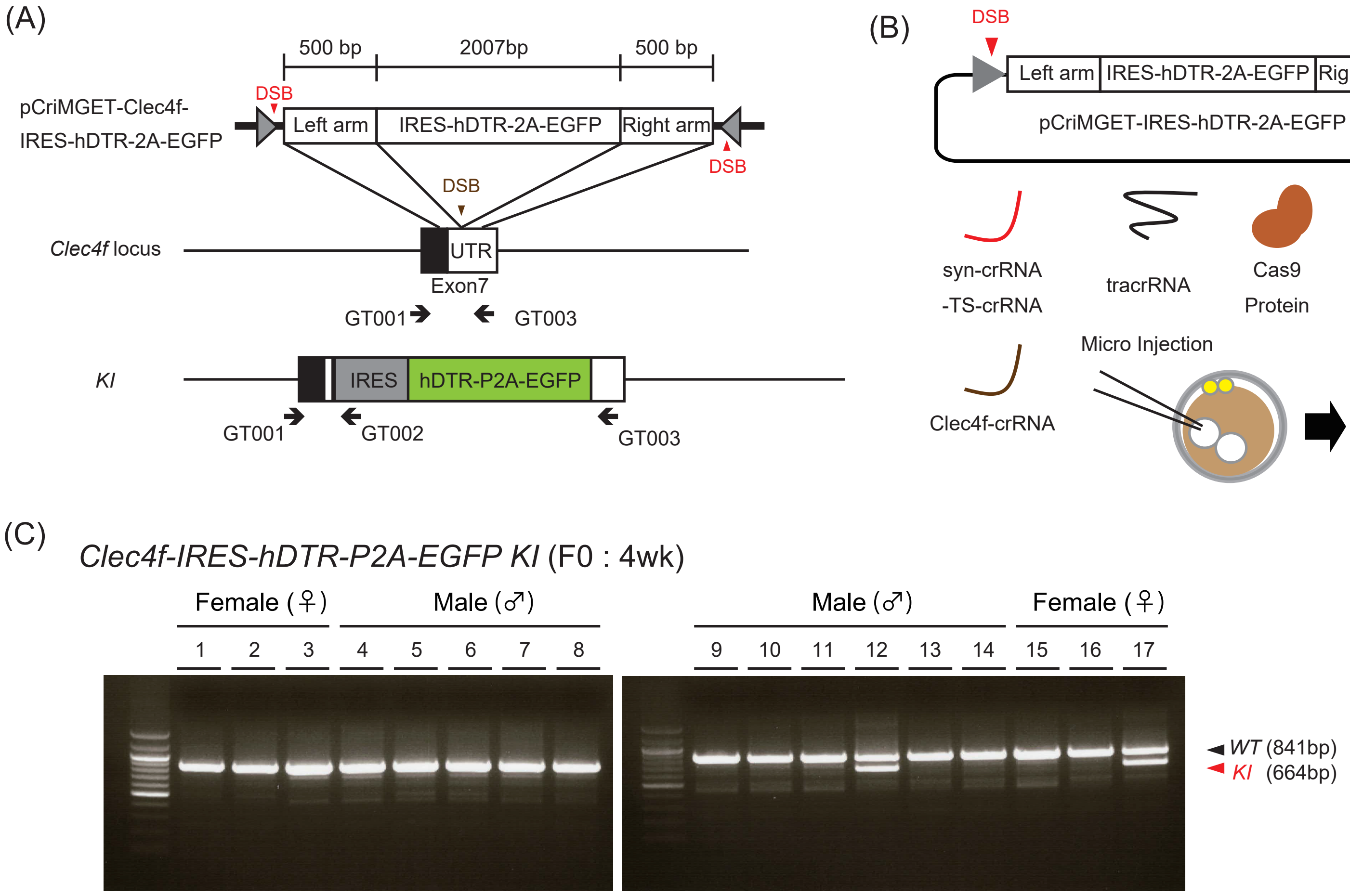

(D)

Total (4wk)	Knock-in Efficiency
17	2 (11.8%)

(E) 5’ Junction

3’ UTR

Left arm — IRES

KI seq. 5’ - tatgttgacctctgaGGCCAGTAGACCAGCA-----//-----TTCAGGGCTTCCTGTCGCCCCTCTCC - 3’

2/2(100%) 5’ - tatgttgacctctgaGGCCAGTAGACCAGCA-----//-----TTCAGGGCTTCCTGTCGCCCCTCTCC - 3’

3’ Junction

3’ UTR

EGFP — Right arm

KI seq. 5’ - GACGAGCTGTACAAGTAAAGCCTGTATTGCCCTG-----//-----CAGGTTTCTGGATctttctcttttc - 3’

2/2(100%) 5’ - GACGAGCTGTACAAGTAAAGCCTGTATTGCCCTG-----//-----CAGGTTTCTGGATctttctcttttc - 3’

Supplementary Figure S8. Generation of *Clec4f-IRES-hDTR-2A-EGFP* knock-in mice via pCriMGET system. (A) Schematic overview of pCriMGET-mediated in-frame knock-in (*KI*) strategy at the *Clec4f* gene locus. *IRES-hDTR -2A-EGFP* transgene was inserted into the 3′-UTR on exon 7 of *Clec4f* gene. (B) Strategy for generating *Clec4f-IRES -hDTR-2A-EGFP* knock-in mice via the pCriMGET system. (C) Genotyping PCR for *Clec4f-IRES-hDTR-2A-EGFP* knock-in mice at 4-week-old. *KI* (664 bp) and *WT* (841 bp) bands used PCR primer sets of GT001/GT002 and GT001/GT003 shown in (A), respectively. (D) Knock-in efficiency in embryos. (E) Sequence analysis of knock-in embryos. PCR products amplified from the 5’ - and 3’ -junction regions from each knock-in embryo were sequenced. Upper- and lower-case letters indicate sequences inside and outside of the donor cassette, respectively.

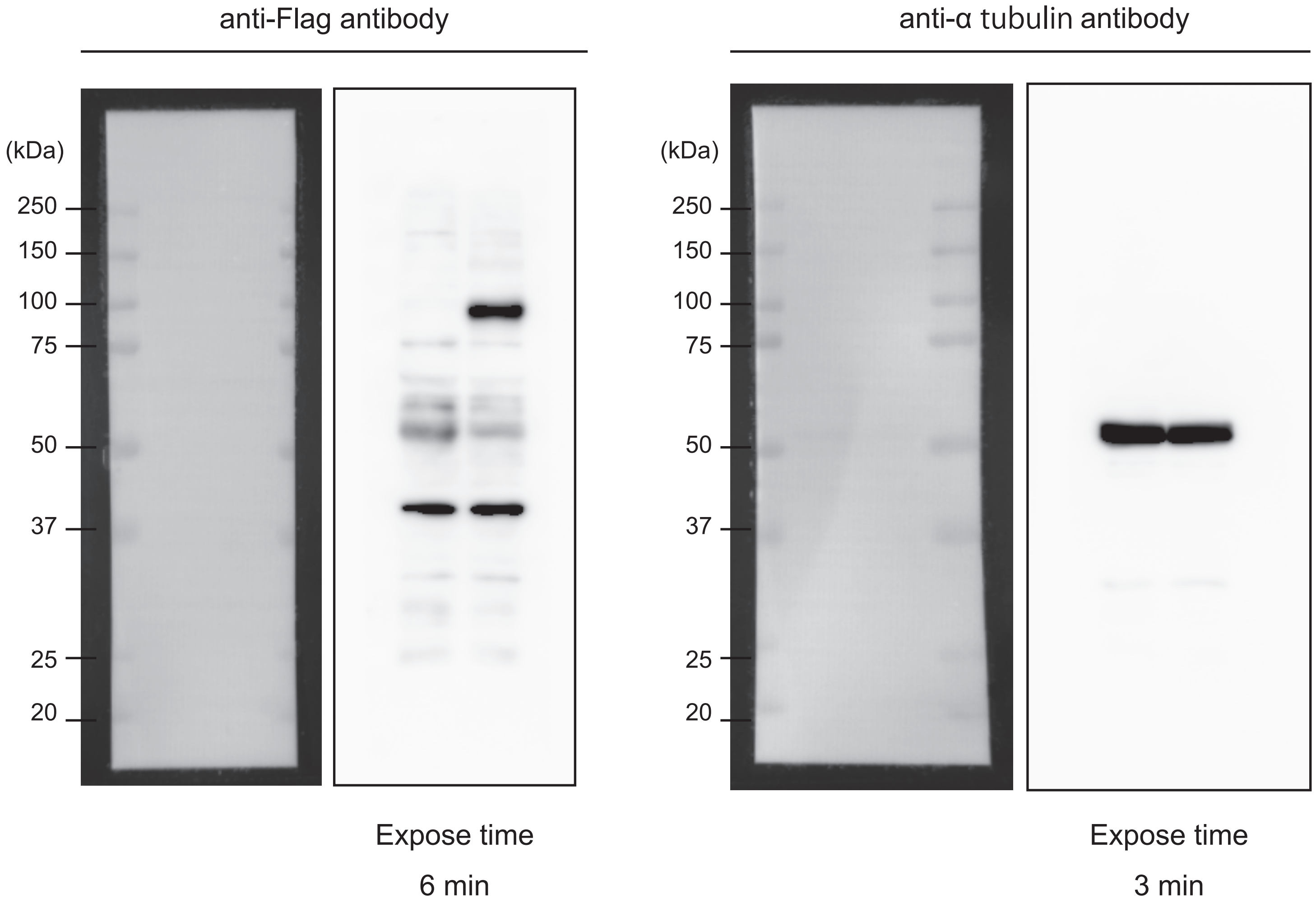

Supplementary Figure S9. Images of the full-length, unprocessed membranes and western blots shown in Fig. 7A.

Supplementary Table S1. off-target analysis of syn-crRNA-TS-crRNA.

Site Name	Sequence	Indel mutation frequency (Mutant / Total)	Position
syn-crRNA-TS	GCTGTCCCCAGTGCATATTCAGG	/	-
CriMGET_OT1	tCTGTCaCCtGTGCATATTCTGG	0 / 10	chr8: 122046509 - 122046531
CriMGET_OT2	GCTGTCCCtAGTGCtTcTTCTGG	0 / 10	chr3: 63846534 - 63846556
CriMGET_OT3	tCTGTCCCCAGTGCtTcTTCTGG	0 / 10	chr5: 125910366 - 125910388
CriMGET_OT4	cCTGTCgCCtGTGCATATTCTGG	0 / 10	chr2: 163966823 - 163966845
CriMGET_OT5	GCTaTCCCCAtTGCAgATTCCGG	0 / 10	chr11: 60913224 - 60913246

The on-target sequence (syn-crRNA-TS) and potential off-target sequences in the mouse genome (OT1–OT5). Mismatched bases are shown in lower case with an underline. PAM sequences are shown in red letters. Genomic DNA loci of OT1–OT5 from every donor gene-integrated embryo (#2, #4, #6, #7, #9, #10, #12, #14, #16, #19 in Figure 6C) were sequenced, and the indel mutation frequency was examined. Position shows the genomic locus of potential off-target site. OT, off-target; /, not tested.

Supplementary Table S2. Oligonucleotides encoding sgRNAs in pX330.

sgRNA name	Direction	Sequence (5' to 3')
syn-crRNA -TS-sgRNA	Sense	CACCGCTGTCCCCAGTGCATATTC
	Anti-sense	AAACGAATATGCACTGGGGACAGC
T2A-sgRNA	Sense	CACCGTGGAGGAGAATCCCGGCCC
	Anti-sense	AAACGGGCCGGGATTCTCCTCCAC
AAVS1-sgRNA	Sense	CACCGGGGCCACTAGGGACAGGAT
	Anti-sense	AAACATCCTGTCCCTAGTGGCCCC

Supplementary Table S3. Oligonucleotides for genotyping and off-target analysis.

Primer name	Direction	Sequence (5' to 3')
CAG-EGFP Tg GT001	F	AGCAGAAGAACGGCATCAAGGTGA
CAG-EGFP Tg GT002	R	AACTCCAGCAGGACCATGTGATCG
Tbx3-3FEG_GT001	F	GAGCCTGTTTCCTTACCCCTACAC
Tbx3-3FEG_GT002	R	GTGGTGCAGATGAACTTCAGGGTC
Tbx3-3FEG_GT003	R	TTGACCAAACTGGAATGGAGAGACC
Tbx3-3FEG_5' arm Fw	F	CTGTCCTCTGGCTCAGTGTCCTTGTCAC
CriMGET_OT1_F	F	CTGGCCGTCCCTTTCCTAGGTTTC
CriMGET_OT1_R	R	AGATGCTTGCCTCCAGCACTGTTT
CriMGET_OT2_F	F	TGCCAATGCAGAGTTCAAACCCCA
CriMGET_OT2_R	R	GAGCCTGTTTCCTTACCCCTACAC
CriMGET_OT3_F	F	GTGGACTGGTGTTCTTTTGCCTGC
CriMGET_OT3_R	R	AGCCACTGCCAATTCTCCTCCTTG
CriMGET_OT4_F	F	CAGGATAACAGGGGCCTCCCAATG
CriMGET_OT4_R	R	GTGGTGCAGATGAACTTCAGGGTC
CriMGET_OT5_F	F	AGTGTCGGTCTGCTGGTACAAACG
CriMGET_OT5_R	R	AACGGTACCGAGCTGCCTTTGTTT
Chr8_F_219-244	F	CTCATGGTGTCAAACTCTGAAGTCCG
Chr8_R_465-490	R	GGGTCAAATCCCTCTTAATGACCAAG
Clec4f-KI_GT001	F	AATGTGTGATGAGTGTGGCATGC
Clec4f-KI_GT002	R	TAACATATAGACAAACGCACACCGG
Clec4f-KI_GT003	R	AGCTGCTGTCTGCACTGTCACTG
mC/EG KI GT001	F	TCGGTCAATACACTACATGGCGTG
mC/EG KI GT002	R	TCTCGTTGGGGTCTTTGCTCAG
mC/EG KI GT003	F	CTCCTTGATGATGGCCATGTTATCC
mC/EG KI GT004	R	AACAGTACGAACGCGCCGAGG
Tbx3-3FEG_GT004	R	CAGTCTTCATAGACAGTTCCACAGGAC
Clec4f-KI_GT004	R	TTAAGGAATGCCATTCCACCCAG
